# An Advanced Human Bone Tissue Culture Model for the Assessment of Implant Osteointegration In Vitro

**DOI:** 10.3390/ijms25105322

**Published:** 2024-05-13

**Authors:** Melania Maglio, Milena Fini, Maria Sartori, Giorgia Codispoti, Veronica Borsari, Dante Dallari, Simone Ambretti, Martina Rocchi, Matilde Tschon

**Affiliations:** 1 IRCCS Istituto Ortopedico Rizzoli, Complex Structure of Surgical Sciences and Technologies, 40136 Bologna, Italy; melania.maglio@ior.it (M.M.); giorgia.codispoti@ior.it (G.C.); veronica.borsari@ior.it (V.B.); matilde.tschon@ior.it (M.T.); 2 IRCCS Istituto Ortopedico Rizzoli, Scientific Direction, 40136 Bologna, Italy; milena.fini@ior.it; 3 IRCCS Istituto Ortopedico Rizzoli, Reconstructive Orthopaedic Surgery and Innovative Techniques—Musculoskeletal Tissue Bank, 40136 Bologna, Italy; dante.dallari@ior.it (D.D.); martina.rocchi@ior.it (M.R.); 4 Microbiology Unit, IRCCS Azienda Ospedaliero—Universitaria di Bologna, 40138 Bologna, Italy; 5 Department of Medical and Surgical Sciences (DIMEC), University of Bologna, 40126 Bologna, Italy

**Keywords:** advanced in vitro model, tissue culture model, osteointegration, alternative methods, human bone

## Abstract

In the field of biomaterials for prosthetic reconstructive surgery, there is the lack of advanced innovative methods to investigate the potentialities of smart biomaterials before in vivo tests. Despite the complex osteointegration process being difficult to recreate in vitro, this study proposes an advanced in vitro tissue culture model of osteointegration using human bone. Cubic samples of trabecular bone were harvested, as waste material, from hip arthroplasty; inner cylindrical defects were created and assigned to the following groups: (1) empty defects (CTRneg); (2) defects implanted with a cytotoxic copper pin (CTRpos); (3) defects implanted with standard titanium pins (Ti). Tissues were dynamically cultured in mini rotating bioreactors and assessed weekly for viability and sterility. After 8 weeks, immunoenzymatic, microtomographic, histological, and histomorphometric analyses were performed. The model was able to simulate the effects of implantation of the materials, showing a drop in viability in CTR+, while Ti appears to have a trophic effect on bone. MicroCT and a histological analysis supported the results, with signs of matrix and bone deposition at the Ti implant site. Data suggest the reliability of the tested model in recreating the osteointegration process in vitro with the aim of reducing and refining in vivo preclinical models.

## 1. Introduction

Biomaterials for orthopaedic implantation have represented a huge innovation, leading to impressive progress in reconstructive surgery for both surgeons and patients [1,2]; biomaterials, scaffolds, and medical devices greatly improve patients’ quality of life, in terms of pain relief, functional recovery to daily and working activities, and healthy aging.

The use of biomaterials for orthopaedic implantation allows for the compensation of mechanical failure of damaged bone (e.g., fractures or oncologic disease) by mimicking the native structure of the tissue. They also favour and enhance bone growth with specific coating and the presence of growth factors. Depending on their permanent or non-permanent use, they either temporarily support the bone microenvironment, in the case of fixation means or degradable devices, or stably remain in the implantation site. The evaluation for the most promising materials, which can blend the necessary mechanical and biological features, has been boosted, especially for the latter condition. Nowadays, the most used implantable devices are metallic, polymeric, or ceramic materials, each showing different characteristics and whose efficiency can vary according to the implantation sites [3]. Ceramic materials (e.g., hydroxyapatite and tricalcium phosphate) can be inert or bioactive, are highly biocompatible, and are easily shaped according to the morphology most suitable for their application, despite being less mechanically performing. Polymers, whether of a natural (e.g., hyaluronic acid, chitosan, silk) or synthetic origin (e.g., PMMA, PEEK, PE), can also be easily processed but non-biodegradable synthetic polymers tend to be biologically inert [4]. Most of the implantable devices used in orthopaedics are made of metals or their alloy (e.g., titanium, Co-Cr), due to their ease of workability, biocompatibility, and high strength and mechanical competence. The possibility of engineering surfaces over time has also improved their biological and osseointegration properties [5].

Osteointegration has been defined as a direct structural and functional connection between ordered, living bone and the implant surface [6]. Osteointegration depends on the biocompatibility, physical, mechanical, and topological characteristics of biomaterials and is critical for implant stability and clinical long-term success [7]. Moreover, implant osteointegration is a complex process that cannot be reduced to the mere growth of new bone in direct contact with the implant and according to its shape, but it also requires compliance with specific mechanical aspects to guarantee implant stability and to avoid relative micromotion with adjacent tissues under functional loads [8,9].

Over time, orthopaedics science has brought the use of biomaterials to a higher level than the simple replacement/fixation of a damaged segment, aligning with the most recent approaches that emphasize tissue repair and regeneration [10]. Despite significant advances in this field, the poor mechanical and biological integrations of an implant in the host bone, as well as the onset of infective processes after implantation, still pose big challenges [11,12]. To overcome these issues, the design of implant material has been implemented with bioactive functionalization, with antimicrobial properties or actively enhancing tissue regeneration and implant integration. These materials also serve as carriers for a controlled delivery of bioactive molecules or drugs [13,14,15,16].

In the modern era, high technological innovations in materials and their manufacturing contribute to the development of intelligent and multifunctional biomaterials, able to interact with the host bone and promote tissue regeneration by inductive and cell- and molecular-mediated effects [16,17,18,19].

Although the use of many implants, including titanium, is a well-established practice in orthopaedics, the continuous implementation in terms of design, micro-architecture, and coatings, to overcome the above-mentioned issues and to address the increasing clinical needs, requires a constantly new preclinical evaluation of biocompatibility and efficacy. Preclinical evaluations are of fundamental importance to detect proper indicators of safety, bioactivity, and osteointegration. However, the possibility to evaluate a human implant failure ex vivo is not so common; thus, osteointegration studies still require the use of in vivo models, which are essential for the preclinical evaluations. Over the years, there has been a growing awareness of the need to expand and improve in vitro models, also in compliance with the principle of the 3 Rs—Reduction, Replacement, and Refinement [20]—to limit the use of in vivo animal models and carry out preliminary phases of the evaluation and selection of materials using in silico models and in vitro cultures. The expansion of the field of in vitro methods has brought notable optimizations in the planning and implementation of in vivo studies. Tests for cytotoxicity, biocompatibility, and bioactivity, as described by international regulations for the in vitro evaluation of new medical devices, have facilitated essential pre-screening of the most promising materials, thereby reducing, and sometimes replacing, the need for in vivo studies. Classic monolayer cell cultures have significantly evolved over the years, thanks to co-cultures, sandwich cultures, use of inserts or scaffolds, microfluidic systems, and 3D printing. These advancements made these study models increasingly complex and versatile. By recreating physiological conditions and using human cells and tissues, researchers have been able to delve deeper into the pathogenetic mechanisms of some pathologies and detail its microenvironment. This has, in turn, facilitated the design of in vivo models, enabling researchers to better focus on study targets, assessments, monitoring, and study conditions, thereby contributing to the refinement of animal studies. However, despite these advancements, it remains challenging to fully recreate the hierarchical structure of bone and mimic its complex biological and mechanical crosstalk [21]. The field of advanced in vitro experimental modelling, relying on humanized 2D co- and tri-cultures or 3D tissue cultures, is of paramount importance, yet it remains significantly limited and the need to test and standardize reliable and repeatable protocols is growing [22].

The aim of the present study was to develop an advanced human tissue culture model to study biological properties and osteointegration in vitro. To evaluate the validity of the results obtained in vitro, a material already commonly used in the clinic was used and compared with a positive cytotoxic control (copper). Preliminary in vitro cytotoxicity testing of these biomaterials was conducted using ISO standard methods. Additionally, the reliability of the 3D model was investigated by comparing the results with standard titanium osteointegration outcomes obtained with an in vivo study in which such material was implanted for 12 weeks.

## 2. Results

### 2.1. In Vitro Cytotoxicity Assessment of Materials

The results are presented in Table 1 as the mean ± SD obtained from three replicates. The Alamar Blue assay showed no significant effect of the Ti material on the viability of the MG-63 cells after 72 h while the Cu material exhibited cytotoxic effects according to ISO 10993-5 [23] as it decreased cell viability by more than 30%. The NR uptake of Ti was higher compared to Cu and CTR pos. Light microscopy observation showed that MG-63 cells in direct contact with Ti specimens were stained by NR vital dye and maintained the same morphology as CTRneg MG-63 cells after 72 h of culture, while the MG-63 cells in contact with Cu rods did not incorporate the NR vital staining showing altered rounded morphology. Finally, LDH activity resulted from damaged cell membranes was as low as CTR neg for cells incubated with Ti while Cu led to higher values than CTRpos. 

### 2.2. Viability Test

**Figure 1:**
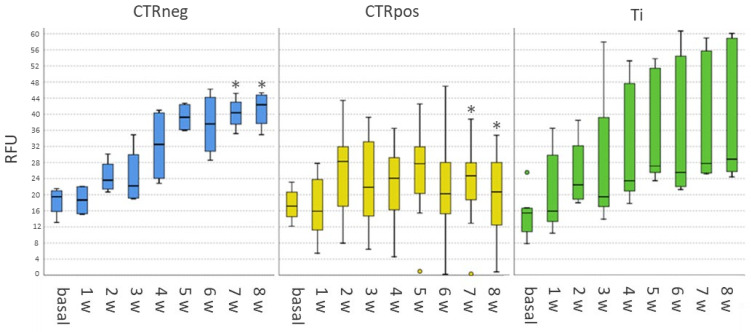
Boxplots of Alamar Blue viability assay results over the 8-week experimental time in the three tissue culture conditions expressed as an RFU (Reference Fluorescent Unit). CTRneg vs. CTRpos at 7 and 8 weeks (*, *p* < 0.05). The Mann–Whitney test with Bonferroni correction.

The results showed that the in vitro 3D tissue culture model remains viable for 8 weeks with viability data for the negative control and Ti constantly increasing, while decreasing for the cytotoxic copper control. At all experimental times, no statistically significant differences were detected between Ti and empty controls. At 7- and 8-week timepoints, viability values of the negative controls were significantly higher than the cytotoxic copper control (*p* < 0.05 at both experimental times). By considering the delta values of viability between 0- and 8-week timepoints, CTRpos had lower values than Ti (*p* < 0.05) and CTRneg (*p* < 0.049) (Figure 1).

### 2.3. Sterility Tests

**Figure 2:**
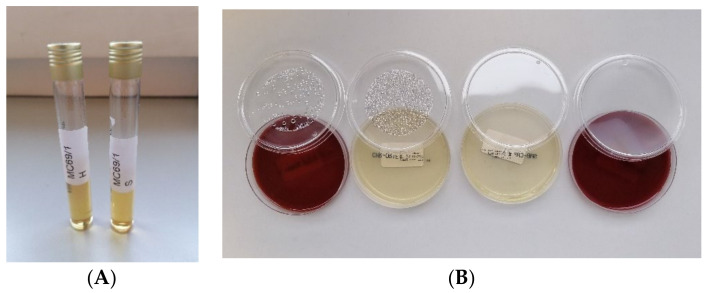
(**A**) Tubes containing TSB and THIO broths inoculated with the tissue culture supernatant. (**B**) Plates inoculated with TSB broth, inoculated with the sample supernatant after 7 days of incubation: from left, blood agar, orientation agar, Sabouraud agar incubated anaerobically, and blood agar incubated anaerobically.

The TSB and THIO broths inoculated with all the supernatants were negative after 7 days of incubation for all timepoints (from 0 to 8 weeks) (Figure 2A), demonstrating the sterility of the tissue culture bone. Seeding in control plates of each broth, inoculated with each supernatant after 7 days of incubation, showed no growth of microorganisms for all experimental times, confirming the sterility of the in vitro manipulation process of bone tissue (Figure 2B).

### 2.4. Immunoenzymatic Results

**Figure 3:**
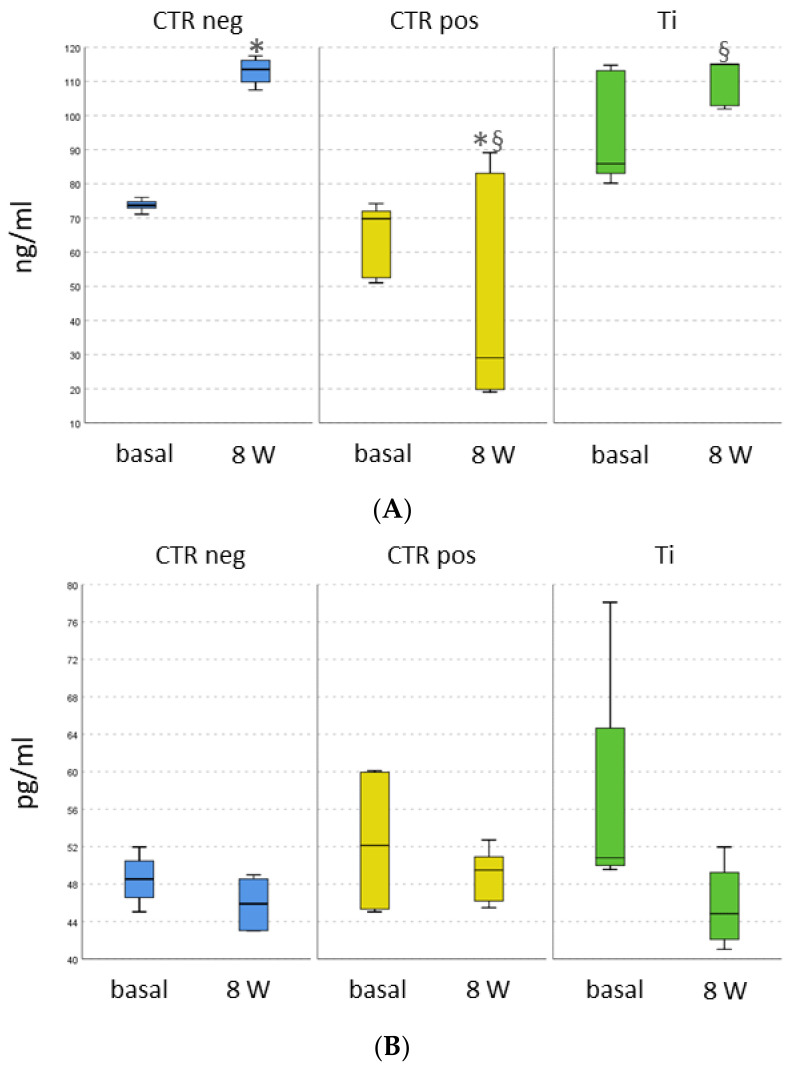
Results of ELISA immunoassays conducted on cell supernatants of 3D osteointegration models at time zero and after 8 weeks of culture. CTRneg: empty bone defects; CTRpos: bone defects with copper implant; Ti: bone defects with titanium alloy implant. (**A**) Osteocalcin, a marker of bone mineralisation; (**B**) interleukin 1 beta, a pro-inflammatory cytokine. The Mann–Whitney test with Bonferroni correction, CTRpos vs. CTR neg (*, *p* < 0.0001) and CTRpos vs. Ti (§, *p* = 0.003).

Protein expressions of osteocalcin and interleukin 1 beta are reported in Figure 3. After 8 weeks of tissue culture, the protein expression of osteocalcin in CTRpos samples was significantly decreased in comparison with CTRneg (*p* < 0.0001) and Ti (*p* = 0.003) (Figure 3).

IL1beta results showed a high level of expression at time 0 in all groups without differences; after 8 weeks of culture, CTRpos induced a stable production of IL1beta, while CTRneg and Ti showed a decrease.

### 2.5. MicroCT Results

**Figure 4:**
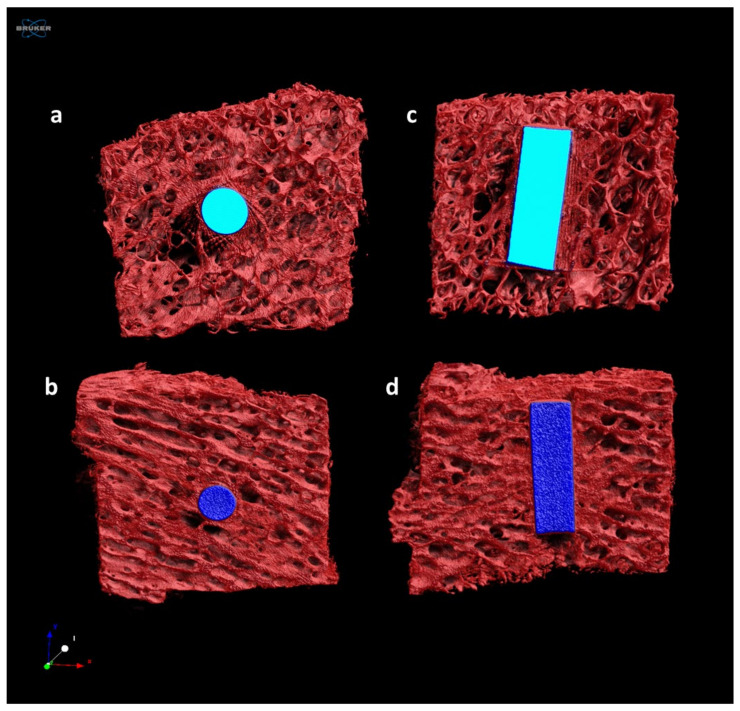
Three-dimensional rendering of representative analysed samples from CTRpos (**a**,**c**) and Ti (**b**,**d**) groups. Transversal (**a**,**b**) and coronal (**c**,**d**) view. Bone (red), copper implant (light blue), titanium implant (dark blue). Multimedia videos are in Supplementary Materials.

The microtomographic images of the samples with metallic implants confirmed the correct positioning of the pins in the created defects (Figure 4). The analyses of the trabecular tissue in the circular VOI identified had the purpose of evaluating the effect of the presence of the Ti material within the bone tissue sample in comparison with the positive control, to highlight whether, in the culture conditions and times adopted, it was possible to detect any alteration at the bone level.

**Figure 5:**
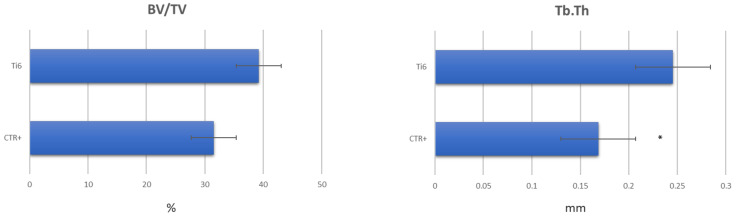
Histograms of 3D morphometric results of CTRpos and Ti samples after 8 weeks of culture. Bootstrap ANOVA: Tb.Th Ti vs. CTRpos (*, *p* < 0.05); CTRpos vs. Ti (*, *p* < 0.05).

A decreasing trend was observed in the BV/TV measurements in the positive control group samples, as well as a significant thinning of the bone trabeculae in comparison with the Ti group in the Tb.Th measurements (*p* < 0.05) (Figure 5).

### 2.6. Histological and Histomorphometric Results

**Figure 6:**
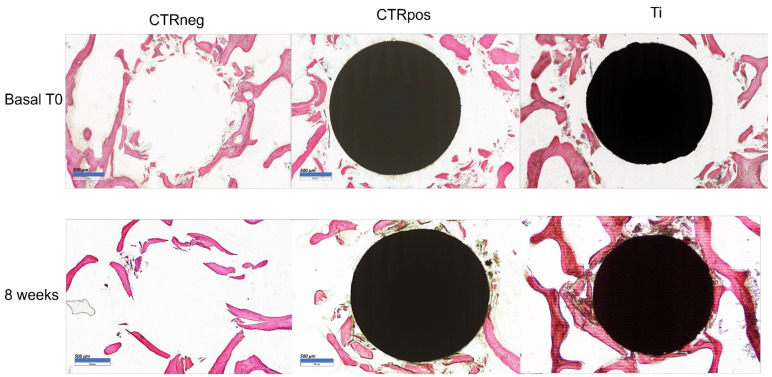
Histological images at the time of implantation (Basal T0, **upper row**) and after 8 weeks of dynamic culture (**lower row**) of the trabecular bone tissue samples—CTR neg: empty defect, CTRpos: cytotoxic copper implant, Ti: standard titanium alloy roughness 6 µm implant. Stevenel Blue–Acid Picrofucsin staining, 4× magnification, bar = 500 µm.

Figure 6 represents histological images of the in vitro 3D tissue models of different groups at the time of implantation and after 8 weeks of culture.

**Figure 7:**
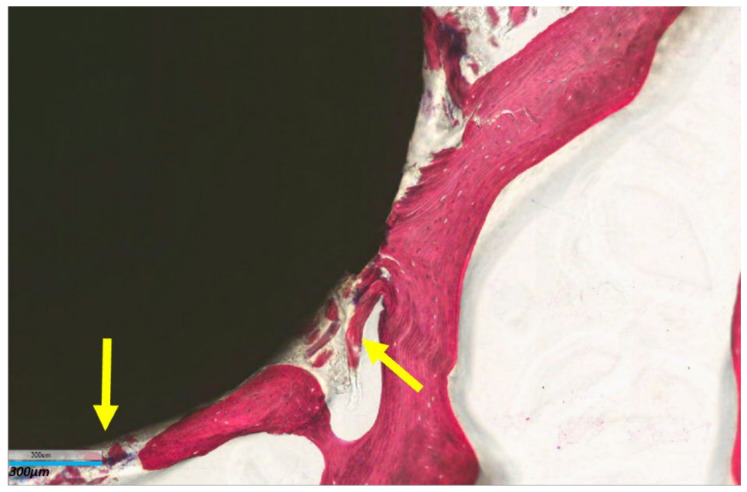
Histological image of trabecular bone tissue sample implanted with Ti alloy at end of 8 weeks of culture (T8). Yellow arrows indicate deposition of new bone tissue trabeculae. Stevenel Blue–Acid Picrofucsin stain, 8× magnification, bar = 300 µm.

Histomorphometric measurements of BIC at the time of implantation and after 8 weeks of dynamic culture are reported in Table 2. Titanium implants induced a significant increase in the bone-to-implant contact after 8 weeks of culture (*p* = 0.006) in comparison with the copper cytotoxic material implanted in the trabecular bone. Similarly, histological images at higher magnification evidenced that the in vitro culture of titanium alloy materials not only maintained the bone tissue sample as viable but also induced bone tissue deposition around the implant (Figure 7).

## 3. Discussion

In the context of the CE marketing, the international reference standard UNI EN ISO 10993 ‘Biological evaluation of medical devices’ [23] serves as a guideline aimed at verifying medical devices’ biocompatibility and safety. Within these standard guidelines, the study of medical devices intended to be implanted in the bone tissue is usually performed in in vivo models since there are no alternative in vitro models for this type of testing. In vivo research enables the simulation of the surgical implantation procedure, monitoring of the animals’ health status over time, observation of any perioperative and postoperative complications, and evaluation of the degree of osteointegration at specified experimental endpoints through histological and histomorphometric assessments. These assessments can be complemented by a 3D imaging analysis and mechanical testing, to provide a comprehensive evaluation of the newly formed bone. However, there is a growing interest in the development of alternative in vitro models driven by ethical, legal, and scientific concerns. This interest stems from the desire to expand reliable screening models for assessing the biocompatibility and performance of materials before their use in vivo and in clinical applications. These in vitro models aim to simulate the biological behaviour of tissues in contact with implant materials in a controlled environment, thereby providing valuable insights into their compatibility and efficacy. 

In the present study, an alternative humanized 3D tissue in vitro model of osteointegration was set up and evaluated for feasibility. This involved the collection of human trabecular samples from an operating room, the creation of small defects, the implantation of standard or cytotoxic materials or none as controls, the maintenance of a long-term culture into mini-bioreactors, and the assessment of main parameters, such as viability, sterility, osteointegration, and bone microarchitecture features. The results obtained from this in vitro model support the hypothesis that this experimental set-up can maintain and even increase (in standard titanium-implanted defects) the trabecular bone viability, support bone matrix deposition, and improve the bone-to-implant contact, in the absence of microbiological contaminations that could affect bone osteointegration. On the other hand, the presence of a known cytotoxic material such as copper resulted in reduced tissue viability, osteocalcin production, and osteointegration measurements.

The assay system demonstrated the ability to recreate an environment resembling the physiological one, as demonstrated by the biological and structural effects observed in the bone tissue in contact with cytotoxic material, which closely resembled those observed in in vivo modelling. In a previous authorized in vivo study of osteointegration, the same standard titanium implant material (with identical 6 µm roughness and dimension) was implanted in the trabecular bone of distal femoral condyles of rabbits for an implantation period of 12 weeks. The histomorphometrically measured bone-to-implant contact after the in vivo implantation was 33.6 ± 12.7; these measurements, taken in a physiologic living body and under mechanical loading conditions, were lined up with those obtained in the present in vitro study after 8 weeks of dynamic culture (28.94 ± 14.29), confirming the validity of the present preliminary data. 

The structural evaluations performed by microCT imaging were somewhat constrained by the presence of metallic implants, particularly copper ones, which produced artifacts that created bias during image segmentation. To mitigate this issue, the decision was made to analyse a tissue crown that was not in direct contact with the material. This approach still enabled an assessment of the bone tissue’s condition in relation to the type of implant present in the cube, thus allowing for an evaluation of the effectiveness of the adopted model.

Another limitation of the present study is the lack of a physiological hormonal regulation, immunological competence, and mechanical loading, all of which are known to affect bone cells and implant osteointegration. These factors represent the complexity of the in vivo system and, when applied to an in vitro model, are simplified to provide a controlled experimental condition [24,25,26,27,28,29]. The focus on osteoimmunology is particularly relevant, considering the growing attention on the influence of the crosstalk of the immune system with bone tissue cells in the osteointegrative processes, even in declinations related to gender and age.

The evaluation of the bone response to a material and, specifically, the degree of the osteointegration of a medical implant device is commonly studied using preclinical animal models because it is a complex orchestration of many interconnected processes, starting from a firm fixation of the implant to bone, followed by protein adhesion and the onset of regenerative processes with an adequate vascular supply and the recruitment of stem cells and growth factors to stimulate cell differentiation towards an osteogenic lineage [30].

To the best of the authors’ knowledge, most of the in vitro studies related to osteointegration properties of a biomaterial are still focused on the use of cell cultures in monolayer or direct and indirect co-cultures seeded onto biomaterials [24,26,31,32,33] to evaluate the effects of tested materials or part of them on viability, morphology, gene/protein expression, and commitment; the set-up of complex tissue cultures mimicking implant osteointegration is still limited. Recently, Zankovic et al. and Przekora et al. performed human bone organ cultures with implanted biomaterials made of 3D-printed Ti6Al4V disks and chitosan/curdlan/hydroxyapatite for 28 and 46 days, respectively, in static conditions [34,35]. In the study by Zankovic et al., although the tested material (TiAlV) is similar to the negative control used in the present study, the in vitro construct utilized an agarose mould, and the bone tissue originated from the tibial plateau. Similarly, in the study by Przekora et al., the evaluations focused on cell viability, as well as the growth and differentiation of osteoblasts, using immunostaining, confocal laser scanning microscopy (CLSM), and scanning electron microscopy (SEM) techniques. The results of these studies confirmed the maintenance of cell viability and proliferation, along with the observation of extracellular matrix deposition. From this perspective, these findings are consistent with those described in the present study, albeit accounting for differences in experimental timelines, materials used, and the type and origin of the analysed tissue. However, the current study uniquely integrated cellular evaluations with microCT imaging, quantitative histology, and, most importantly, comparison with control groups, enhancing the comprehensiveness and reliability of the findings.

To achieve these aims, the present advanced in vitro model was the first to perform a 56-day-long culture under dynamic conditions. It was set up from human bone samples, thereby bridging the gap between the in vivo and human situations, reducing animal experimentation, and including all bone cellular phenotypes and the extracellular matrix in a tri-dimensional configuration. Moreover, in comparison with 2D cultures, the 3D model of osteointegration avoids the risk of cell dedifferentiation, allows for long-term cultures, retains extracellular matrix components and secreted growth factors, preserves native structural organization and configuration, and gives the possibility to perform histological and biomechanical evaluations. This humanized 3D model has the potential to provide valuable insights necessary for bridging the gap between in vitro and in vivo [29], and future improvements would include (i) the mechanical stimulation of bone explants by means of bioreactors; (ii) addition of exogenous hormonal substances to the media; (iii) integration with already existing in vitro monoculture models, for example, with macrophagic cell lines, to study the influence of osteoimmunology; (iv) collection of bone samples from patients affected by different bone diseases and pathologies (such as patients with osteoporosis or diabetes); and (v) evaluation of sex and gender determinants that might affect osteointegration. The model can undergo validation with other materials already established in clinical practice, such as ceramics, polymers, or composites. Furthermore, it can be utilized to assess the same type of material with and without surface modifications, such as coatings or engineering enhancements, aimed at improving its performance in osteointegration. Additionally, depending on the experimental set-up, further analyses can be conducted. These may include immunostainings to evaluate cellular responses and interactions within the tissue model. Furthermore, mechanical and structural characterizations can be performed to assess the biomechanical properties and structural integrity of the implanted materials and the surrounding bone tissue. These comprehensive analyses will provide a deeper understanding of the behaviour and performance of the materials in the context of osteointegration, thereby contributing to advancements in biomaterial design and clinical application.

## 4. Materials and Methods

### 4.1. In Vitro Cytotoxicity Assessment of Materials

Titanium alloy rods (Ti) with a surface roughness of 6 µm (manufactured by ZARE S.r.l, Boretto (RE), Italy) were used in this study. The rods were 2 mm in diameter and 6 mm in length. Additionally, copper wires (Merck cod. 326429, Merck, Darmstadt, Germay) measuring 2 mm in diameter and 6 mm in length were included. These materials were subjected to in vitro cytotoxicity testing following the international standard guidelines outlined in ‘UNI EN ISO 10993—Part 5 (2009) Tests for in vitro cytotoxicity’ [23].

Human MG-63 cells were expanded in a DMEM medium (Sigma-Aldrich Corp., St. Louis, MO, USA), with 10% FBS (Lonza, Euroclone, Milan, Italy), 2 mM L-glutamine, and 0.1 mg/mL of penicillin–streptomycin, at 37 °C in a humidified atmosphere with 5% CO_2_. Cells were detached at 85–90% confluence, counted, and seeded at the final concentration of 2 × 10^4^ cell/cm^2^ in 12-well plates for 24 h. Then, Ti and copper (Cu) rods were placed in direct contact with the cell monolayer. MG-63 cells with no biomaterials served as a negative control (CTRneg) for an incubation period of 72 h, while MG-63 cells treated with 0.5% phenol in the medium served as a positive control (CTRpos). 

An Alamar Blue assay (Thermo Fisher Scientific, Waltham, MA, USA) was used to quantify the viability of MG-63 cells at 72 hrs. Briefly, Alamar Blue Cell Viability Reagent was added (1:10 *v*/*v* with culture medium) to the cultures for 3.5 h at 37 °C: viable cells internalize and reduce non-fluorescent Resazurin to fluorescent Resorufin. Then, fluorescence was read at 530ex–590emnm wavelengths by a micro-plate reader (VICTOR X2030, Perkin Elmer, Milan, Italy) and normalized to the values obtained for CTRneg. At the same experimental time, MG-63 cells were stained with a 0.33% Neutral Red (NR) solution (Sigma-Aldrich, St. Louis, MI, USA) to evaluate cell morphology and measure living cells. Finally, the activity of Lactic Dehydrogenase (LDH) released from damaged cells was measured using the Cytotoxicity Detection Kit (Roche Diagnostics GmbH Roche Applied Science, Mannheim, Germany), according to the manufacturer’s instructions.

The results of the Alamar blue test and NR uptake values are reported as a percentage of CTRneg. LDH results are reported as a percentage of cytotoxicity calculated using the equation

(1) $$Cytotoxity\;(\%)=\frac{exp\;value-low\;ctr}{high\;ctr-low\;ctr}\times 100$$

### 4.2. Trabecular Bone Specimen Harvesting

This study was approved by the Comitato Etico Indipendente Area Vasta Emilia Centro (829/2019/Sper/IOR) and was carried out in accordance with relevant guidelines and regulations (IRCCS Istituto Ortopedico Rizzoli has kept the ISO 9001 [36] quality certification since 2008, with special reference to the research area). Written informed consent was obtained from all individual participants included in this study and undergoing total hip arthroplasty. Eligibility criteria were an age between 18 and 75 years and capability to provide written informed consent. Exclusion criteria were the presence of infections (HBV, HIV, HCV), haematological disorders, pregnancy or lactation, and any musculoskeletal pathologies affecting bone.

**Figure 8:**
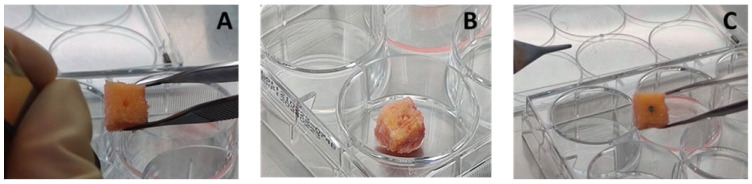
The set-up of the 3D in vitro models: a central defect (2 mm in diameter) was created in the human trabecular bone specimens—(**A**) a negative empty defect (CTR−); (**B**) implanted with positive cytotoxic copper wire (CTR+); (**C**) implanted with a titanium alloy pin with a surface roughness of 6 μm (Ti).

Trabecular bone specimens were aseptically obtained from femoral heads and excised as waste material from 4 patients with a mean age of 63.5 ± 10.1 years (range: 52–72) to obtain a total number of 21 cubic (1.5 × 1.5 ± 0.1 cm) samples of bone. By using a 2 mm surgical drill, cylindrical defects were aseptically created, as shown in Figure 8A; after measuring weights, the 3D tissue specimens were randomly assigned to the following groups of treatment:

- Negative controls (CTRneg): cylindrical defects left empty;
- Positive controls (CTRpos): cylindrical defects press fit implanted with a well-known cytotoxic material (copper wires, Merck cod. 326429, 2 mm in diameter and 6 mm in length) (Figure 8B);
- The 6 µm rough titanium alloy (Ti): cylindrical defects press fit implanted with standard titanium pins (ZARE S.r.l, Boretto (RE), Italy), 2 mm in diameter, 6 mm in length, and a 6 µm surface roughness (Figure 8C).

All specimens were cultured in DMEM (Dulbecco’s Modified Eagle’s Medium, Sigma Aldrich, Merck) with 10% Foetal Bovine Serum (Lonza, Euroclone, Pero, Italy), 100 U/mL of penicillin, and 100 µg/mL of streptomycin in mini rotating bioreactors (Mini-Bioreactor Tube Spin, Merck Life Science, Darmstadt, Germany) in standard culture conditions (5% CO_2_, 21% O_2_, 37 °C). Three-dimensional tissue models were cultured for 8 weeks with the medium changed twice a week.

At time 0 (before pin implantation) and at each week timepoint, viability and sterility analyses were performed. Immunoenzymatic, histological, and histomorphometric analyses were conducted at time 0 and at the final 8-week timepoint, while microtomographic analyses were conducted at the final timepoint. 

### 4.3. Viability Test on 3D Tissue Model

The Alamar Blue test (Thermo Fisher Scientific, Waltham, MA, USA) has been used for the quantitative determination of cell viability at the time of implantation and at the following experimental times [25,26]. Briefly, Alamar Blue Cell Viability Reagent was added (1:10 *v*/*v* with culture medium) to the 3D tissue models for 3.5 h at 37 °C: viable cells internalize and reduce non-fluorescent Resazurin to fluorescent Resorufin. Then, fluorescence was read at 530ex–590emnm wavelengths by a micro-plate reader (VICTOR X2030, Perkin Elmer, Italy) and normalized with specimen weights and expressed as an RFU (Reference Fluorescence Unit). 

### 4.4. Sterility Test

Sterility of the human 3D models was assessed at the time of surgery, before pin implantation, and, thereafter, at each 7-day-interval timepoint. Two millilitres of the medium was aseptically harvested and cultured in Tryptic Soy Broth (TSB) and Thyoglicolate Broth (THIO) (Liofilchem, Roseto degli Abruzzi, Italia) for the determination of bacterial or fungal growth. Cultures were checked every day for turbidity and, after a 7-day culture at 37 °C, 0.5 mL of each culture was plated in blood agar, CHROMagar Orientation, and Sabouraud Dextrose Agar with Chloramphenicol (Vacutest KIMA, Arzergrande, Italy) and cultured at 37 °C in aerobic and anaerobic conditions. Plates were read for differential bacterial growth after 24, 48, and 72 h. 

### 4.5. Immunoenzymatic Tests

At time zero and after 8 weeks of culture, cell supernatants were collected, which, after centrifugation to remove particulate matter, were frozen at −80 °C and used for the quantification of osteocalcin (human OST, Raybiotech ELISA assay) and interleukin 1 beta (human IL-1 beta, 4A BIOTECH ELISA assay) by means of enzyme immunoassays. 

### 4.6. Microtomographic Analysis

Samples were scanned with the microCt system Skyscan 1172 (Bruker microCT, Kontich, Belgium) at a source voltage of 100 kV and a source current of 100 μA, using an aluminium + cuprum filter, at a nominal resolution set at 12 μm. Samples were rotated 360°, with a rotation step of 0.3° and a frame averaging of 6. The reconstruction of the obtained dataset was performed with NRecon software (version 1.7.4.6, Bruker microCT), applying corrections for specific misalignment, ring artifact reduction, and beam hardening.

**Figure 9:**
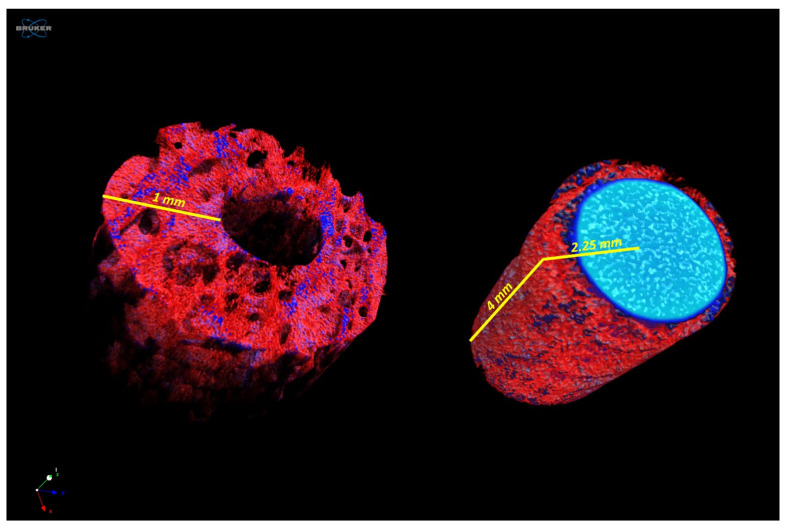
The identification of the VOI for the morphometric micro-CT analysis: on the left, the analysed VOI correspondent to the bone around the implant/peri-implant area until 1 mm in the radial direction; on the right, the VOI of the metallic implant and peri-implant bone until 2.25 mm in the radial direction, excluded by the analysed dataset.

CTAn software (version 1.20.3.0; Bruker Micro-CT, Belgium) was used for quantitative 3D analyses in a volume of interest (VOI) corresponding to the peripheral crown (4 mm height) built from 2.25 mm in the radial direction from the centre of the implant and extended for 1 mm (Figure 9)

The following morphological parameters were evaluated:

- Bone density BV/TV (%), expressed as the ratio between the volume of the peri-implant trabecular bone and the total volume of the VOI;
- Trabecular Thickness Tb.Th (in mm), calculated in a model-independent manner described by Hildebrand and Ruegsegger on the entire VOI.

### 4.7. Histological and Histomorphometric Analyses

Specimens at time 0 and after 8 weeks of dynamic culture were fixed in a 4%-paraformaldehyde-buffered solution (Sigma Aldrich, St. Louis, MO, USA) for three days, extensively washed, and processed undecalcified for resin embedding. Briefly, samples were dehydrated in increasing ethanol concentrations of 48 h each (50%, 70%, two steps in 95%, and two steps in 100% for 24 h) and embedded in polymethylmethacrylate (PMMA) (Methacrylate; Merck, Shuchardt, Hohenbrunn, Germany). After polymerization, resin blocks were cut according to a transversal and longitudinal plan with a microtome (Leica SP1600 Leica Microsystems Spa), thinned, and polished up to a 50 ± 10 μm thickness by a grinding and polishing machine (Saphir 550, ATM). Histological slides were stained with Stevenel Blue-Picrofucsin (Merck, Darmstadt, Germay) and images have been acquired with a high-resolution scanner for digital pathology (Aperio Scanscope CS System, Aperio Technologies, Vista, CA, USA) at the maximum resolution (1781 × 1467 pixels). For each material, three sections have been evaluated to assess the osteointegrative process, measuring the bone-to-implant contact (BIC, %), expressed as the ratio between the perimeter of the material and the bone contact perimeter. 

### 4.8. Statistical Analyses 

Each statistical analysis was performed using SPSS v.19.0 (IBM Corp., Armonk, NY, USA). The Shapiro–Wilk test was performed to test normality of continuous variables. The Levene test was used to assess the homoscedasticity of the data. The ANOVA test was performed to assess the between-group differences of continuous, normally distributed, and homoscedastic data, and the Mann–Whitney non-parametric test was used otherwise. The ANOVA test, followed by the post hoc Sidak test for pairwise comparisons, was performed to assess the among-group differences of continuous, normally distributed, and homoscedastic data, and the Kruskal–Wallis non-parametric test, followed by the post hoc Mann–Whitney test with Bonferroni correction for multiple comparisons, was used otherwise. With a small sample size, the ANOVA test was evaluated using Bootstrap methods using Bootstrap R package lmboot. For all tests, *p* < 0.05 was considered significant.

## 5. Conclusions

The model described serves as a versatile and representative tool for evaluating the in vitro osseointegration of implants intended for orthopaedic use. Its adaptability allows for testing with multiple materials and recreating various pathological conditions, making it a valuable platform for exploring alternative methods in orthopaedics. By offering the capability to mimic different clinical scenarios and assess the performance of diverse implant materials, this model paves the way for the development and validation of novel orthopaedic implants and treatment strategies. Its versatility and ability to replicate complex physiological and pathological conditions make it an essential tool for advancing orthopaedic research and ultimately improving patient outcomes.

## Figures and Tables

**Table 1:** Results of the in vitro cytotoxicity assessments performed according to ISO 10993-5 [23]; **, *p* < 0.005; ***, *p* < 0.0005). ANOVA with post hoc Sidak test—Alamar Blue and NR uptake: Ti vs. Cu and CTRpos (*p* < 0.0005); Cu vs. CTRpos (*p* < 0.005); LDH: Cu vs. Ti and CTRpos (*p* < 0.0005); CTRpos vs. Ti (*p* < 0.0005).

	Ti	Cu	CTRpos
Alamar Blue test (%)	96.47 ± 1.35 ***	0.37 ± 0.09 **	0
NR uptake (%)	89.22 ± 2.29 ***	5.83 ± 0.68 **	0.59 ± 0.06
LDH (%)	0	141.46 ± 1.87 ***	100.00 ± 10.61 ***

**Table 2:** Histomorphometric results of BIC percentage measures on stained slices at the implantation time (Basal T0) and after 8 weeks of culture (T8). CTRpos: trabecular bone with cytotoxic copper implant; Ti: trabecular bone with standard titanium alloy roughness 6 µm implant. Data are expressed as mean and SD. Mann–Whitney test: **, *p* < 0.005.

BIC (%)	CTRpos	Ti
Basal T0	0.00 ± 0.00	1.33 ± 1.33
T8	10.22 ± 5.57	28.94 ± 14.29 **

## Data Availability

The data presented in this study are available on request from the corresponding author.

## Supplementary Materials

The following supporting information can be downloaded at: https://www.mdpi.com/article/10.3390/ijms25105322/s1.

## Abbreviations

BIC	Bone-to-implant contact
BV/TV	Bone volume/total volume
Co-Cr	Cobalt-chrome
Cu	Copper
IL.1 beta	Interleukin 1 beta
microCT	Microtomography
CTRneg	Negative control
OST	Osteocalcin
PEEK	Polyether ether ketone
PE	Polyethylene
PMMA	Polymethylmethacrylate
CTRpos	Positive control
THIO	Thyoglicolate broth
Ti	Titanium
Tb.Th	Trabecular thickness
TSB	Tryptic soy broth
VOI	Volume of interest
